# The acute effect of two exercise modalities on neurocognitive responses in postmenopausal women: A randomized controlled trial

**DOI:** 10.1113/EP092537

**Published:** 2025-05-31

**Authors:** Morgane Le Bourvellec, Nathalie Delpech, Laurent Bosquet, Geoffroy Boucard, Carina Enea

**Affiliations:** ^1^ Laboratoire MOVE (UR20296), Faculté des Sciences du Sport Université de Poitiers Poitiers France; ^2^ Centre de Recherches sur la Cognition et l'Apprentissage Université de Tours, Université de Poitiers, CNRS Tours, Poitiers France

**Keywords:** cognition, exercise, menopause

## Abstract

Menopause‐related cognitive decline, often worsened by vasomotor symptoms (VMS), might be mitigated by high cardiorespiratory fitness (CRF). Although acute exercise supports neurocognitive function, its effects vary by exercise and individual characteristics. In this study, we investigated the acute effects of isometric resistance exercise (IRE) and high‐intensity interval exercise (HIIE) on prefrontal cortex oxygenation and cognitive performance in postmenopausal women and examined the influence of VMS and CRF on these outcomes. A cross‐over randomized controlled trial was conducted among 29 women aged 55 ± 3 years. The HIIE session included two sets of 12 × 15 s at 100% maximal aerobic power, and the IRE session included 4 × 2 min at 30% maximal voluntary force. Cognitive functions were evaluated before and after sessions using the MEM‐III story recall test (episodic memory), Stroop task (inhibitory control) and n‐back task (working memory). Prefrontal cortex oxygenation was assessed by measuring oxyhaemoglobin (ΔHbO_2_), deoxygenated haemoglobin (ΔHHb) and total haemoglobin (ΔtHb) concentrations before, during and after each session. No effect of exercise was noted on cognitive performance. However, prefrontal cortex oxygenation increased during HIIE (ΔHbO_2_: *d* = 0.99, *p* *<* 0.0001; ΔHHb: *d* = 0.68, *p* = 0.018; ΔtHb: *d* = 0.96, *p* = 0.001), during IRE (ΔHbO_2_: *d* = 1.2, *p* = 0.003) and post‐HIIE (ΔHbO_2_ and ΔtHb: *d* > 1; *p* < 0.0001) versus control. CRF positively modulated cognitive and cerebrovascular responses to IRE, whereas VMS showed no influence. IRE and HIIE did not improve cognitive performance in postmenopausal women, but increased prefrontal cortex oxygenation, with sustained effects after HIIE. CRF positively modulated responses, whereas VMS did not, underscoring the importance of maintaining high CRF to support brain health in this population.

## Abstract (highlights)

- **What is the central question of this study?**
  Acute exercise, including isometric resistance exercise and high‐intensity interval aerobic exercise, enhances prefrontal cortex oxygenation and cognitive performance in young adults, with cardiovascular health as a key modulator. However, their effectiveness in postmenopausal women remains uncertain, particularly concerning the influence of vasomotor symptoms and cardiorespiratory fitness on these responses.
- **What is the main finding and its importance?**
  Isometric resistance exercise and high‐intensity interval aerobic exercise do not improve cognitive performance, but do increase prefrontal cortex oxygenation, with sustained effects after high‐intensity interval aerobic exercise. Cardiorespiratory fitness positively modulates postexercise cognitive and cerebrovascular responses, and vasomotor symptoms have no effect.

## 1 INTRODUCTION

Cognitive disorders (or cognitive decline) refer to impairments in one or more cognitive functions, such as memory or executive functions, resulting from brain disease, injury, ageing or other neurological conditions (McDonald, 2017). Globally, they affect >57 million people worldwide, with women disproportionately affected [female‐to‐male ratio = 1.69 (95% confidence interval 1.64–1.73)] (GBD 2019 Dementia Forecasting Collaborators, 2022). In postmenopausal women, the decline in female sex hormones is linked to cognitive impairments in different domains, such as episodic memory and executive functions (e.g. working memory and inhibitory control) (Conde et al., 2021). Oestrogens play a crucial role in supporting synaptic plasticity, neural connectivity and grey matter volume, essentials for cognitive processes (Russell et al., 2019). For instance, their loss following oophorectomy is associated with an increased risk of cognitive decline (Georgakis et al., 2019), and early use of oestrogen therapy can offer neuroprotective effects (Nerattini et al., 2023). Additionally, menopause is associated with vascular changes, including increased blood pressure (Samargandy et al., 2022), arterial stiffness (Samargandy et al., 2020) and endothelial dysfunction (Moreau et al., 2012), which can disrupt cerebrovascular function (Barnes & Corkery, 2018) and impair cerebral perfusion (Guo et al., 2024), contributing to the cognitive decline observed during this stage. Longitudinal studies indicate that peri‐ and postmenopausal women exhibit poorer cognitive performance than premenopausal women, particularly in episodic memory tasks (Greendale et al., 2009; Kilpi et al., 2020) and occasionally in working memory (Maki et al., 2021; Weber et al., 2013). Furthermore, vasomotor symptoms (VMS) appear to exacerbate cognitive decline, with studies showing a negative correlation between the frequency of VMS and episodic memory performance, linked to impairments in hippocampal and prefrontal cortex function (Maki et al., 2008, 2020). Although the exact physiological mechanisms linking VMS to high‐level cognitive functions remain poorly understood, evidence suggests that women experiencing VMS tend to have poorer cardiovascular health, characterized by elevated blood pressure, endothelial dysfunction and increased arterial stiffness (Lee et al., 2022; Yang et al., 2017). These vascular impairments might contribute to cerebrovascular dysfunction, compromising cerebral perfusion and increasing the risk of cognitive decline (Barnes & Corkery, 2018).

One component of cardiovascular health is cardiorespiratory fitness (CRF), which can be modified by physical activity. Regular physical activity is widely recommended for the prevention of neurocognitive disorders across populations (Erickson et al., 2019) and has demonstrated benefits in postmenopausal women (Anderson et al., 2014). The positive effects of physical activity on brain health might be explained by the CRF hypothesis (Agbangla et al., 2019), which suggests that higher CRF improves brain vascularization and oxygenation (Salzman et al., 2022). Furthermore, physical activity promotes the release of neurotrophic factors, such as brain‐derived neurotrophic factor and insulin‐like growth factor‐1, which support hippocampal neurogenesis, synaptogenesis and synaptic plasticity (Cabral et al., 2019). These neurovascular adaptations help to optimize brain function (Moore et al., 2022), thereby enhancing cognitive performance (Colcombe & Kramer, 2003; Rigdon & Loprinzi, 2019). In addition to chronic benefits, acute exercise can also produce immediate cognitive improvements, visible as soon as the exercise session ends (Basso & Suzuki, 2017; Chang et al., 2012). These short‐term effects might arise from physiological changes, such as increased cerebral blood flow, the release of brain‐derived neurotrophic factor and enhanced connectivity within neural circuits (Herold et al., 2019; Pontifex et al., 2019). When exercise is performed regularly, these acute benefits can lead to long‐term neurocognitive adaptations (Voss et al., 2020). The effects of a single exercise session have shown promise in improving cognitive performance in episodic memory (Loprinzi et al., 2019) and executive functions tasks (Huang et al., 2022; Oberste et al., 2019) in the general population. However, few studies have focused specifically on middle‐aged adults (Pontifex et al., 2019), often combining data from both men and women, despite evidence suggesting that sex differences might influence the mechanisms and effectiveness of exercise (Barha et al., 2017; Cortes & De Miguel, 2022).

Recently, two emerging exercise modalities have gained attention for their neurocognitive and cardiovascular benefits, both acute (after a single session) and chronic (after a sustained training programme). High‐intensity interval exercise (HIIE) training has proved effective in improving CRF in postmenopausal women (Ballesta‐García et al., 2020), and its positive effects on cognitive function have been demonstrated in young adults, both acutely (Sudo et al., 2022) and chronically (Alves et al., 2021). Likewise, isometric resistance exercise (IRE) training has shown promise as a highly effective modality for reducing blood pressure in the general population (Cornelissen & Smart, 2013), and its acute and chronic benefits for cognitive functions are also emerging (Zhu et al., 2022), although further exploration in postmenopausal women is needed.

The aims of this randomized controlled crossover study were as follows: (1) to investigate the effects of two exercise modalities (IRE and HIIE) on episodic memory and executive functions and prefrontal cortex oxygenation, with a focus on changes in total haemoglobin concentration as an indicator of variations in cerebral blood flow in postmenopausal women; and (2) to assess whether neurocognitive responses to exercise are influenced by the severity of VMS or by the level of CRF.

## 2 MATERIALS AND METHODS

### 2.1 Ethical approval

This randomized controlled crossover study, conducted from May 2023 to January 2024 at the MOVE laboratory, University of Poitiers, was approved by the French ethics committee (CPP: 2023‐A00284‐41) and registered with the CNIL (MR‐001: 2228637). All participants provided written informed consent prior to inclusion and received an €80 gift card upon completion. The study conformed to the standards set by the latest revision of the *Declaration of Helsinki*. The study protocol is registered on ClinicalTrials.gov (NCT06533982) and adheres to the CONSORT guidelines for randomized controlled trials.

### 2.2 Population and eligibility criteria

Inclusion criteria were as follows: (1) naturally postmenopausal women (>12 months of amenorrhoea and blood estradiol levels < 20 pg mL^−1^ and follicle stimulating hormone > 40 mUI mL^−1^); (2) menopausal for ≤10 years; (3) with or without VMS assessed by the Menopause Rating Scale (MRS) questionnaire. Non‐inclusion criteria were as follows: (1) premature ovarian insufficiency; (2) cardiovascular or respiratory conditions (medically treated hypertension, coronary artery disease, valvular heart disease, arrhythmias, unstable asthma, respiratory failure or pulmonary hypertension); (3) chronic kidney disease; (4) body mass index ≥ 40 kg m^−2^; (5) auditory or visual impairments preventing reading or colour discrimination; (6) recent central neurological or psychiatric disorders (<1 year); and (7) Montreal Cognitive Assessment (MoCA) < 18.

### 2.3 Initial data collection

#### 2.3.1 Characteristics of the women

A global assessment of cognitive function was conducted at the inclusion using the MoCA, a screening tool for neurocognitive impairments (Nasreddine et al., 2005).

The characteristics of participants collected included age, years since menopause, number of children, educational level, daily tobacco and alcohol consumption (yes/no), and moderate‐to‐vigorous physical activity time assessed using the Global Physical Activity Questionnaire (Bull et al., 2009).

Body composition analysis was performed using the *Tanita BC‐418 MA* impedance scale, to assess body weight (in kilograms), body mass index, body fat (in kilograms and as a percentage of body mass) and fat‐free mass (in kilograms).

The severity of menopausal symptoms was evaluated using the MRS and the Hot Flash Related Daily Interference Scale questionnaires (Cavadias et al., 2023; Heinemann et al., 2003).

CRF was assessed through a maximal cardiopulmonary test performed on cycle ergometer using an incremental protocol, supervised by a cardiologist. The test started at 30 W, increasing by 15 W min^−1^, with a cadence of 60 r.p.m., until maximal capacity was reached. The test measured maximal oxygen consumption (${\dot{V}}_{O_{2}\max}$), maximal aerobic power and maximal heart rate (HR_max_).

### 2.4 Classification of groups

The response to the first question of the MRS questionnaire allowed for the classification of women into two groups: women without moderate to severe VMS (score 0–1; VMS−) and women with moderate to severe VMS (score 2–4; VMS+). Additionally, CRF was used to separate the population: those with a ${\dot{V}}_{O_{2}\max}$ ≤ 37.5 mL kg^−1^ min^−1^ of lean body mass (CRF−) and those with a ${\dot{V}}_{O_{2}\max}$ > 37.5 mL kg^−1^ min^−1^ (CRF+).

### 2.5 Intervention sessions

**FIGURE 1:**
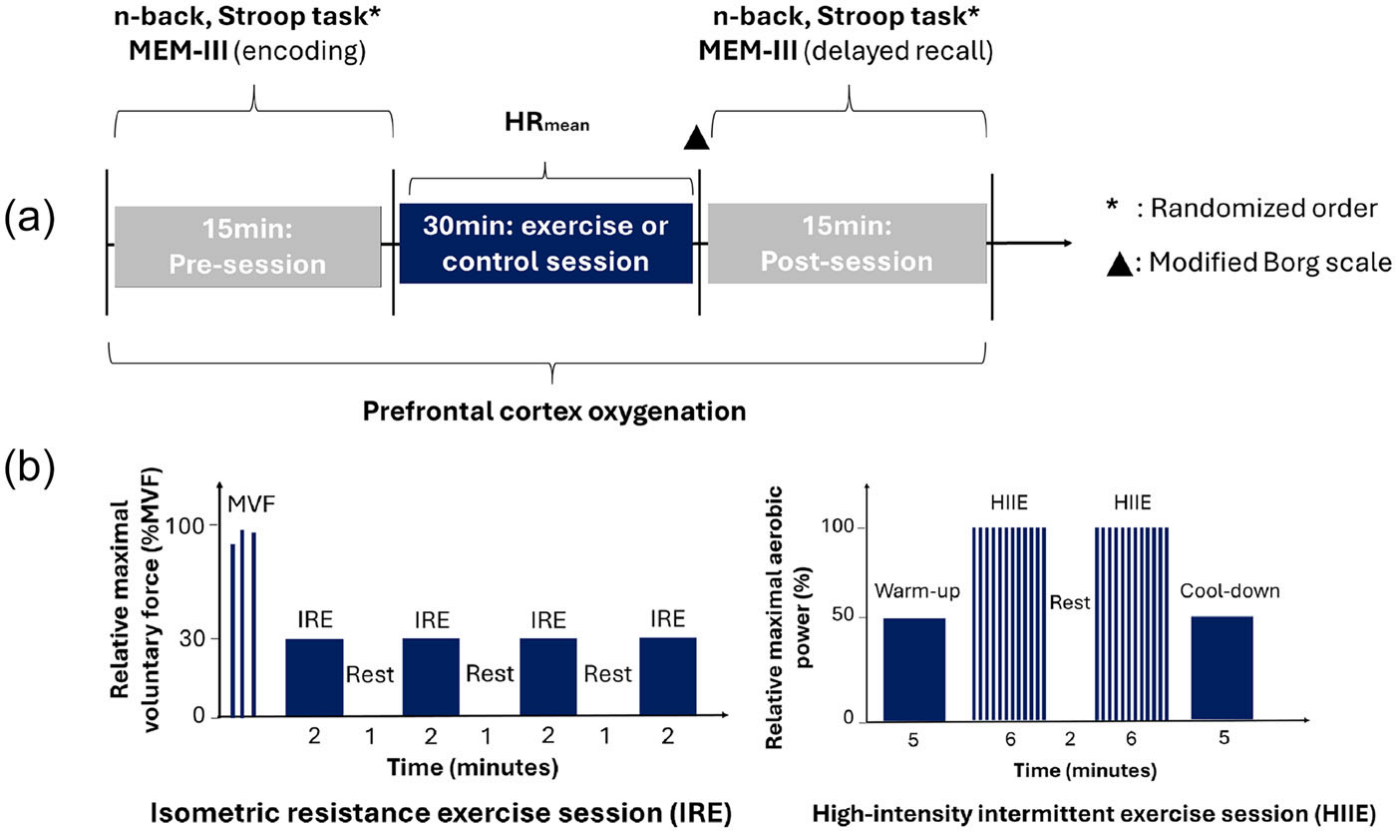
Intervention sessions and neurocognitive measurements. (a) General structure of intervention sessions and measurements. (b) Breakdown of HIIE and IRE sessions. Abbreviations: HIIE, high‐intensity interval exercise session; HR_mean_, mean heart rate; IRE, isometric resistance exercise session; MVF, maximal voluntary force.

On average, experimental sessions took place at 13.30 h ± 3 h. Each participant completed their three sessions at the same time of day, in similar conditions (e.g. on workdays or days off), with a minimal interval of 48 h between sessions. Participants were instructed to refrain from physical activity for 24 h prior to each session. Each session lasted ∼60 min and included cognitive assessments before and after the exercise or control sessions, along with continuous monitoring of prefrontal cortex oxygenation. Heart rate (HR) was recorded using a Polar H10 sensor connected to the Polar Beat app, and perceived exertion was assessed using the modified Borg scale (Figure 1). The order of the sessions was randomized into one of six possible sequences, the order of the cognitive tests (n‐back and Stroop task) into one of two possible orders, and the order of the stories (MEM‐III: memory tasks derived from the Wechsler Memory Scale, Third Edition) into one of three possible sequences, using the ‘Randomizer’ mobile application.

#### 2.5.1 Isometric resistance exercise session

The IRE session involved gripping a handgrip (K‐Force grip, KINVENT) with the dominant hand, which was connected to a tablet displaying the exerted force. Participants were seated with their feet flat on the ground, elbows at 90° and forearms in a neutral position. To begin, they determined their maximal voluntary force (MVF) through three tests, each followed by a 50 s rest interval. The highest value from these trials was used to set the target intensity for the session. The exercise protocol consisted of four 2 min repetitions at 30% of MVF, with 1 min rest intervals between each. Intensity was monitored continuously using visual and auditory feedback from the K‐Force app (Figure 1).

#### 2.5.2 High‐intensity intermittent aerobic exercise session

The HIIE session was performed on a cycloergometer, with intensity calibrated based on the baseline maximal aerobic power of each participant. The session began with a 5 min warm‐up and cool‐down at 50% of maximal aerobic power. The main exercise consisted of two sets of 12 repetitions, each lasting 15 s at 100% of the maximal aerobic power, followed by 15 s passive recovery intervals. A 2 min passive recovery was included between sets (Figure 1).

#### 2.5.3 Control session

The control (CONT) session involved 30 min of seated rest. During this period, participants were allowed to read, use their telephones or converse with the evaluator, but were instructed not to stand or walk. The activity was self‐selected, with the instruction to choose an activity requiring minimal cognitive load.

### 2.6 Cognitive performance evaluations

#### 2.6.1 Episodic memory

The MEM‐III story recall task, derived from the Wechsler Memory Scale‐Revised, was used to assess episodic memory performance (Wechsler, 2001). During the task, the evaluator read a short story consisting of 25 elements to participants twice for memorization prior the CONT, IRE and HIIE sessions. Immediately after the first reading, participants were instructed to recall the story details, constituting the immediate recall score (I1). After the second reading, participants recalled the story details again, constituting the second immediate recall score (I2). Finally, after the CONT, IRE or HIIE sessions, participants were asked to recall the memorized elements, constituting the delayed recall score (D), reflecting episodic memory performance. The scores are calculated out of 25 based on the number of story elements recalled correctly, with reference to a checklist. Three distinct stories were used across the three sessions in a randomized order.

#### 2.6.2 Inhibitory control

The Stroop task was used to assess inhibitory control (Golden, 1978). The task involved reading three boards: Board A, which displayed colour words written in black ink; Board B, which featured coloured crosses for participants to identify; and Board C, which presented colour words written in incongruent coloured ink, requiring participants to identify the ink colour rather than the written word. Scores for each board were recorded based on the number of colours correctly read or identified within 45 s. An interference score (*I*) was then calculated to assess participants’ inhibition abilities, using the formula: *I* = *C* − *C*_predicted_, where *C* is the total number of correct responses on Board C, and *C*_predicted_ is the predicted correct responses based on performance on Board A and Board B. The value of *C*_predicted_ was calculated using the formula: *C*_predicted _= (*A* × *B*)/(*A* + *B*), where *A* and *B* represent the scores on Board A and Board B, respectively.

#### 2.6.3 Working memory

The n‐back task (2‐back version) was used to assess the updating capacity of working memory (Kirchner, 1958). During the task, the experimenter orally presented a sequence of letters, and participants were required to indicate whether the current letter matched the one presented two letters prior by responding ‘yes’ or ‘no.’ Performance was evaluated based on the number of correct responses (score out of 28).

### 2.7 Prefrontal cortex oxygenation

The oxygenation of the right and left prefrontal cortex, measured by concentrations of oxyhaemoglobin (ΔHbO_2_), deoxygenated haemoglobin (ΔHHb) and total haemoglobin (ΔtHb) relative to baseline values, was recorded continuously using near‐infrared spectroscopy (NIRS). The optodes were placed over the Fp1 and Fp2 regions of the prefrontal cortex according to the international EEG 10–20 system. This assessment was performed with the multichannel OxyMon MK‐III device (Artinis Medical Systems, Elst, The Netherlands), connected to the OxySoft signal analysis software (v.3.0), which was calibrated before each use. Baseline signal values corresponded to the average of the last minute of the rest period preceding the cognitive tasks at the start of the sessions. The analysis was completed by M.L.B.

### 2.8 Statistical analyses

The differences between sessions were analysed using repeated‐measures multivariate ANOVAs, incorporating repeated factors for time (pre‐ and post‐session within each session) and session type (CONT, IRE and HIIE). Between‐subject effects included the severity of vasomotor symptoms (VMS+ vs. VMS−) and CRF (CRF− vs. CRF+). If the assumption of sphericity was violated (assessed by Mauchly's test), degrees of freedom (d.f.) were adjusted using Greenhouse–Geisser correction for epsilon values <0.75, or Huynh–Feldt correction for values >0.75. Bonferroni *post hoc* tests were conducted when ANOVA results were significant. The effect size of the observed differences was interpreted using Cohen's *d* (0.2 < *d* < 0.5, small effect; 0.5 < *d* < 0.8, medium effect; *d* > 0.8, large effect). A 5% α risk was applied for statistical analyses. All continuous data are presented as means ± SD or medians (interquartile range), depending on the normality of the data distribution assessed by the Shapiro–Wilk test. Categorical data are presented as the number of observations (percentages). Statistical analyses were performed using JASP software (v.0.16.4).

## 3 RESULTS

### 3.1 Population

**FIGURE 2:**
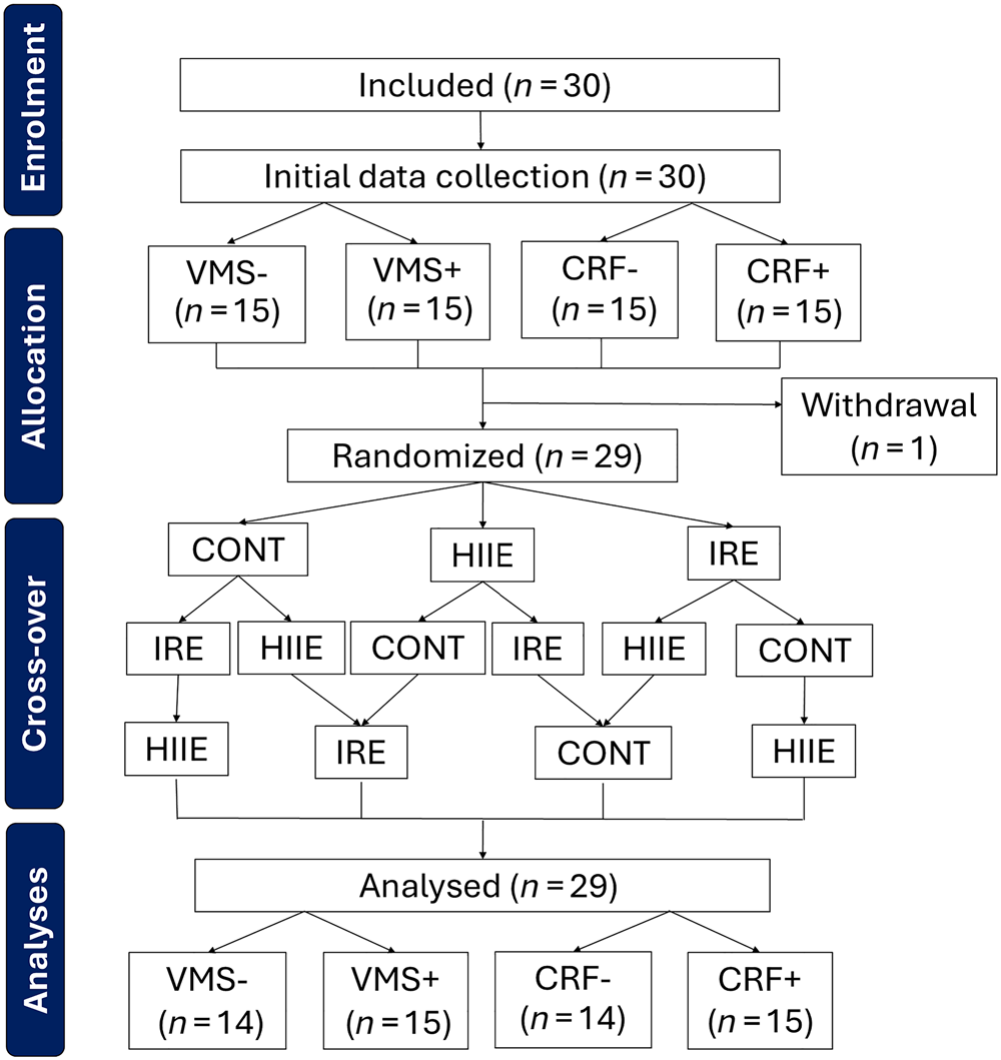
Flow chart of the study. Abbreviations: CONT, control session; CRF−, ${\dot{V}}_{O_{2}\max}$ ≤37.5 mL kg^−1^ min^−1^; CRF+, ${\dot{V}}_{O_{2}\max}$ > 37.5 mL kg^−1^ min^−1^; HIIE, high‐intensity aerobic intermittent exercise session; IRE, isometric resistance exercise session; VMS−, without moderate to severe vasomotor symptoms; VMS+, with moderate to severe vasomotor symptoms.

Thirty women were initially included in the study; however, one participant withdrew, resulting in a final sample of 29 women (*n* = 14 VMS− and *n* = 15 VMS+; *n* = 14 with ${\dot{V}}_{O_{2}\max}$ ≤ 37.5 mL kg^−1^ min^−1^ and *n* = 15 with ${\dot{V}}_{O_{2}\max}$ > 37.5 mL kg^−1^ min^−1^), as shown in Figure 2. The participants had a mean age of 55 ± 3 years and had been menopausal for 3.1 ± 2.4 years. Their mean MoCA score was 27.7 ± 1.2 (range 26–30), and none were using menopausal hormone therapy. The characteristics of the 29 women, both overall and stratified by classification (VMS−/VMS+; CRF−/CRF+), are presented in Table 1.

**TABLE 1:** Population characteristics of postmenopausal women.

Characteristic	Total (*n* = 29)	VMS− (*n* = 14)	VMS+ (*n* = 15)	CRF− (*n* = 14)	CRF+ (*n* = 15)
**Population characteristics**					
Age (years)	55 ± 3	56 ± 4	55 ± 3	57 ± 3	55 ± 4
Years post menopause (FMP)	3.1 ± 2.4	4.1 ± 2.8	2.3 ± 1.2	2.9 ± 1.9	3.4 ± 2.7
Child (*n*)	2 (0)	2 (0)	2 (0)	2 (0)	2 (0)
Educational level [*n* (%)]					
Secondary education	6 (20)	3 (21)	3 (20)	4 (29)	2 (13)
High school graduate	7 (24)	4 (29)	3 (20)	4 (29)	3 (20)
Bachelor degree	9 (32)	6 (43)	3 (20)	3 (21)	6 (40)
≥Master degree	7 (24)	1 (7)	6 (40)	3 (21)	4 (27)
MoCA	27.7 ± 1.2	27.6 ± 1.0	27.7 ± 1.3	27.6 ± 1.2	27.7 ± 1.2
**Body composition**					
BMI (kg/m^2^)	22.8 ± 4.5	23.8 ± 4.9	21.8 ± 4.1	24.3 ± 5.3	21.4 ± 3.0
Fat mass (kg)	19.9 ± 8.7	21.4 ± 10	18.6 ± 7.4	22.8 ± 10.5	17.3 ± 5.9
Lean mass (kg)	42.1 ± 5.1	42.3 ± 6.3	41.9 ± 3.8	44.1 ± 5.3	40.2 ± 4.2^†^
Percentage fat mass	31 ± 7.4	32.4 ± 8.1	29.8 ± 6.7	32.6 ± 7.7	29.6 ± 6.9
**Physical capacity**					
${\dot{V}}_{O_{2}\max}$ lean (mL kg^−1^ min^−1^)^a^	37.9 ± 7.1	37.6 ± 7.4	38.1 ± 7.1	32.4 ± 3.8	43.0 ± 5.1^††^
${\dot{V}}_{O_{2}\max}$ (mL kg^−1^ min^−1^)	25.8 ± 6.3	25.6 ± 6.4	26.2 ± 6.6	21.3 ± 2.7	30.7 ± 5.3^†††^
Maximal aerobic power (W)	145 ± 25	144 ± 32	146 ± 17	131 ± 18	158 ± 23^†††^
HR_max_ (beats min^−1^)	160 ± 10	161 ± 9	159 ± 10	159 ± 11	161 ± 9
MVF (kg)	24.7 ± 3.9	24.3 ± 4.2	25.1 ± 3.7	24.1 ± 4.3	25.3 ± 3.6
**Symptoms**					
MRS	14 (5)	12.5 (6.5)	15 (5.5)^*^	16 (5.3)	12 (3.5)
HFRDIS	15 (33)	3 (8.75)	34 (28.5)^**^	16.5 (42.5)	15 (27.5)
**Lifestyle**					
MVPA (min week^−1^)	300 (360)	360 (514)	270 (242)	270 (330)	338 (365)
Smoke [*n* (%)]	3 (10%)	3 (20%)	0 (0%)	3 (21%)	0 (0%)
Alcohol [*n* (%)]	1 (3%)	0 (0%)	1 (7%)	0 (0%)	1 (7%)

*Note*: Continuous data are presented as means ± SD or medians (interquartile range) and categorical data as number of observations (percentages). Abbreviations: BMI, body mass index; CRF−, ${\dot{V}}_{O_{2}\max}$ lean ≤37.5 mL kg^−1^ min^−1^; CRF+, ${\dot{V}}_{O_{2}\max}$ lean > 37.5 mL kg^−1^ min^−1^; FMP, final menstrual period; HFRDIS, Hot Flash Related Daily Interference Scale; HR_max_, maximum heart rate; MRS, Menopause Rating Scale; MVF, maximum voluntary force; MVPA, moderate to vigorous physical activity. VMS−, without moderate to severe vasomotor symptoms; VMS+, with moderate to severe vasomotor symptoms. Differences between VMS groups: ^*^ *p =* 0.039; ^**^ *p <* 0.0001. Difference between CRF groups: ^†^ *p =* 0.034; ^††^ *p =* 0.001; ^†††^ *p <* 0.0001. To compare groups, Student's and Mann–Whitney *U*‐tests were performed for continuous data, the χ^2^ test for categorical data.

^a^ ${\dot{V}}_{O_{2}\max}$ was calculated from lean mass.

### 3.2 Sessions

The exercise sessions were scheduled on average 10 ± 6 days apart, and no adverse effects were reported during or after the sessions. The perceived difficulty of the exercise, as measured by the modified Borg scale, was 5 ± 2 for the IRE session and 4 ± 2 for the HIIE session. The average heart rate during the sessions was 74 ± 10 beats min^−1^ for IRE and 122 ± 13 beats min^−1^ for HIIE.

**FIGURE 3:**
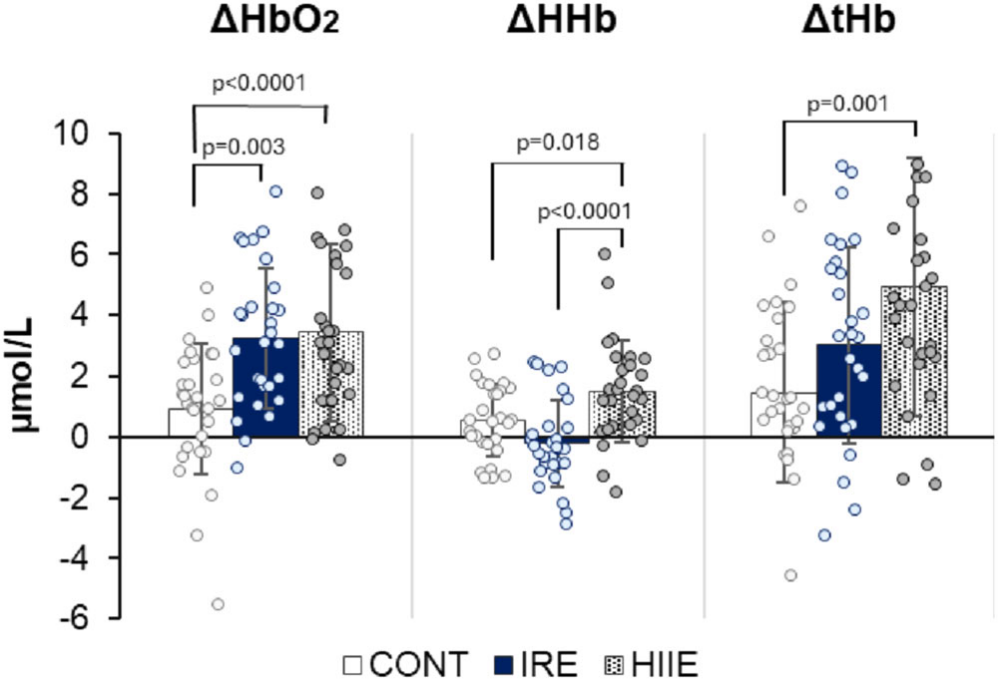
Prefrontal cortex oxygenation during the sessions CONT, IRE and HIIE. *n* = 29. Abbreviations: CONT, control session; HIIE, high‐intensity aerobic intermittent exercise session; IRE, isometric resistance exercise session; ΔHbO_2_, oxyhaemoglobin; ΔHHb, deoxyhaemoglobin; ΔtHb, total haemoglobin.

Significant differences in prefrontal cortex oxygenation levels were observed across the different sessions [ΔHbO_2_: *F*(2) = 9.16, *p <* 0.0001; ΔHHb: *F*(2) = 12.1, *p <* 0.0001; ΔtHb: *F*(2) = 7.4, *p =* 0.002]. The HIIE session showed higher prefrontal cortex oxygenation than the CONT session for all three parameters (ΔHbO_2_: *d* = 0.99, *p <* 0.0001; ΔHHb: *d* = 0.68, *p =* 0.018; ΔtHb: *d* = 0.96, *p =* 0.001). Additionally, ΔHHb levels were higher during the HIIE session compared with the IRE session (*d* = 1.2, *p <* 0.0001), whereas ΔHbO_2_ values were higher during the IRE session than during the CONT session (*d* = 0.89, *p =* 0.003) (Figure 3).

### 3.3 Effect of exercise modalities on neurocognitive responses to exercise

#### 3.3.1 Cognitive performance

Regarding cognitive performance, repeated‐measures ANOVA revealed a positive time effect on the interference score [Stroop task: *F*(2) = 10.3, *p =* 0.004, *d* = 0.3] and on episodic memory (MEM‐III: *F*(2) = 166.2, *p <* 0.0001; I2 vs. D: *p =* 0.002, *d* = 0.3) across the three sessions, with no significant session × time interaction observed. No differences were found between sessions for the correct number of responses in the n‐back task and for the delayed recall in the MEM‐III story task. The results are presented in Table 2.

**TABLE 2:** Cognitive performance across the sessions CONT, IRE and HIIE.

Test	CONT (*n* = 29)	IRE (*n* = 29)	HIIE (*n* = 29)	Repeated‐measures ANOVA		
				Time	Session	Time × session
n‐back						
Pre	24.9 ± 1.8	24.4 ± 1.9	25.3 ± 1.6	*F*(1) = 0.9	*F*(2) = 2.5	*F*(2) = 1.8
Post	24.6 ± 1.5	24.7 ± 1.7	24.9 ± 1.6	*p =* 0.359	*p =* 0.096	*p =* 0.181
Stroop						
Pre	1.0 ± 8.6	2.1 ± 8.7	0.6 ± 7.9	***F*(1) = 10.3**	*F*(2) = 0.1	*F*(2) = 0.9
Post	3.0 ± 7.9	3.2 ± 10.2	4.5 ± 7.6	***p =* 0.004**	*p =* 0.882	*p =* 0.397
MEM‐III						
Pre (I1)	15.9 ± 3.5	16.3 ± 3.5	15.8 ± 3.7	***F*(2) = 166.2**	*F*(2) = 0.9	*F*(4) = 0.4
Pre (I2)	19.9 ± 3.1	20.5 ± 2.6	19.9 ± 3.1	***p <* 0.0001**	*p =* 0.432	*p =* 0.797
Post (D)	19.2 ± 3.5	19.8 ± 2.6	18.8 ± 2.6			

Abbreviations: CONT, control session; HIIE, high‐intensity aerobic intermittent exercise session; IRE, isometric resistance exercise session.

#### 3.3.2 Prefrontal cortex oxygenation during cognitive tasks

**FIGURE 4:**
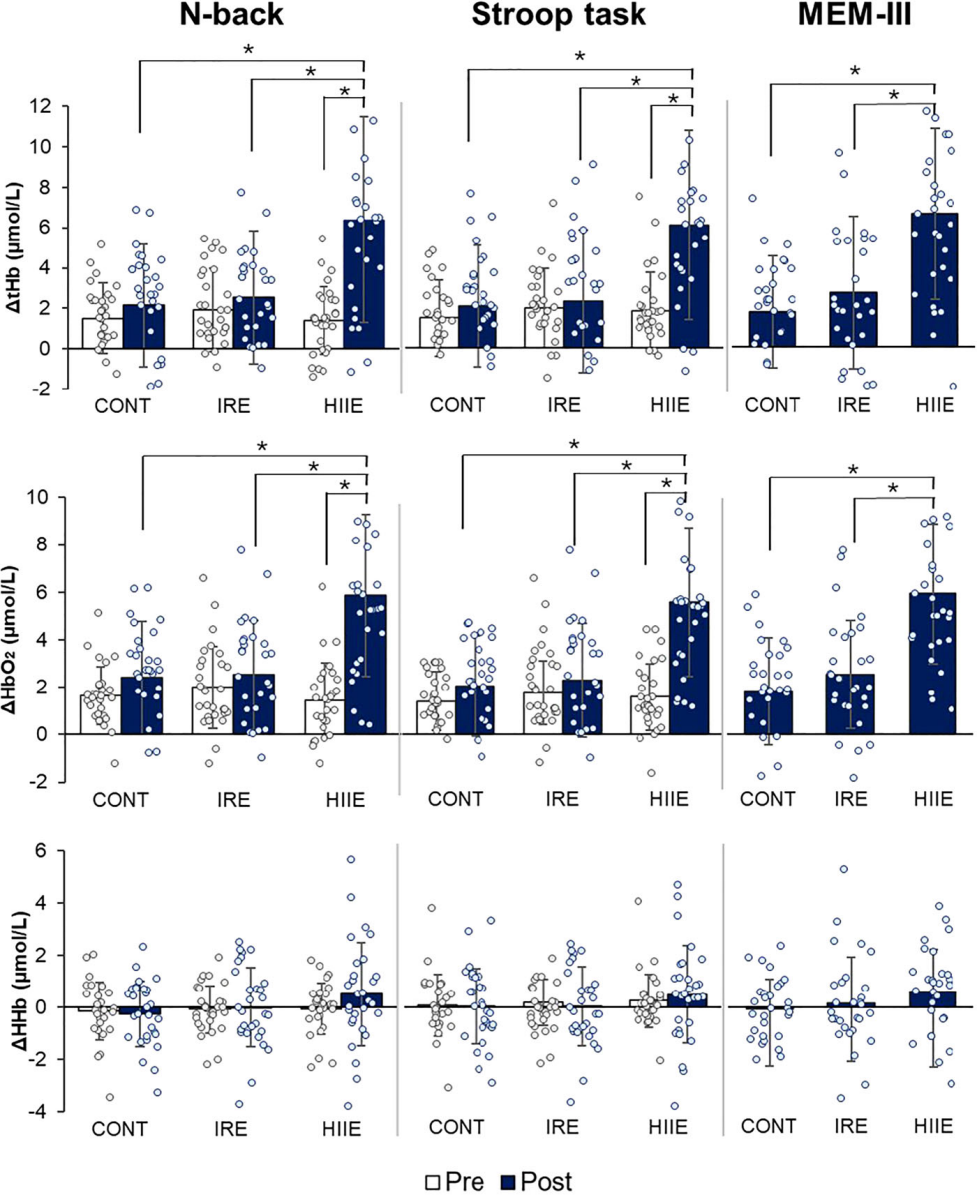
Prefrontal oxygenation during cognitive tasks. ^*^ *p* < 0.0001; *n* = 29. Abbreviations: CONT, control session; HIIE, high‐intensity aerobic intermittent exercise session; IRE, isometric resistance exercise session; ΔHbO_2_, oxyhaemoglobin; ΔHHb, deoxyhaemoglobin; ΔtHb, total haemoglobin.

Concerning ΔtHb concentrations during cognitive tasks, a session effect was observed across all three tests [n‐back: *F*(2) = 5.9, *p =* 0.005; Stroop: *F*(2) = 9.2, *p <* 0.0001; MEM‐III: *F*(2) = 12.9, *p <* 0.0001]. Additionally, a time effect was noted for the n‐back test [*F*(1) = 24.0, *p <* 0.0001] and the Stroop task [*F*(1) = 19.2, *p <* 0.0001], with a session × time interaction effect [n‐back: *F*(2) = 18.7, *p <* 0.0001; Stroop: *F*(1.6) = 11.4, *p <* 0.0001]. *Post hoc* analyses revealed that ΔtHb levels were significantly higher following the HIIE session compared with baseline [n‐back: *d* = 1.6, *p <* 0.0001; Stroop: *d* = 1.4, *p <* 0.0001] and in comparison to ΔtHb concentrations measured during the three cognitive tasks after both the IRE session (n‐back: *d* = 1.3, *p <* 0.0001; Stroop: *d* = 1.3, *p <* 0.0001; MEM‐III: *d* = 1.0, *p <* 0.0001) and the CONT session (n‐back: *d* = 1.3, *P <* 0.0001; Stroop: *d* = 1.3, *p <* 0.0001; MEM‐III: *d* = 1.2, *p <* 0.0001). These findings are presented in Figure 4.

A similar effect was observed for ΔHbO_2_ concentrations as for ΔtHb. A session effect was found for all three tests: n‐back [*F*(2) = 7.5, *p =* 0.002], Stroop [*F*(2) = 12.1, *p <* 0.0001] and MEM‐III [*F*(2) = 21.1, *p <* 0.0001]. Additionally, a time effect was observed for the n‐back test [*F*(1) = 39.2, *p <* 0.0001] and the Stroop task [*F*(1) = 46.2, *p <* 0.0001], with a session × time interaction effect [n‐back: *F*(2) = 18.7, *p <* 0.0001; Stroop: *F*(1.7) = 18.5, *p <* 0.0001]. The ΔHbO_2_ values were also significantly higher after the HIIE session compared with baseline (n‐back: *d* = 1.9, *p <* 0.0001; Stroop: *d* = 1.9, *p <* 0.0001) and compared with values obtained after the IRE session (n‐back: *d* = 1.5, *p <* 0.0001; Stroop: *d* = 1.6, *p <* 0.0001; MEM‐III: *d* = 1.3, *p <* 0.0001) and CONT session (n‐back: *d* = 1.5, *p <* 0.0001; Stroop: *d* = 1.6, *p <* 0.0001; MEM‐III: *d* = 1.5, *p <* 0.0001). These results are shown in Figure 4.

However, no effect of session [n‐back: *F*(2) = 1.3, *p =* 0.295; Stroop: *F*(2) = 1.8, *p =* 0.185; MEM‐III: *F*(2) = 0.88, *p =* 0.422] or time [n‐back: *F*(2) = 0.95, *p =* 0.339; Stroop: *F*(2) = 0.06, *p =* 0.802] was observed on ΔHHb concentrations during the three cognitive tasks (Figure 4).

### 3.4 Effect of VMS and CRF on neurocognitive responses to exercise

#### 3.4.1 Effect of VMS

Regarding cerebrovascular responses to exercise, no effect of VMS was noted during the sessions for ΔtHb [*F*(1) = 0.22, *p =* 0.642], ΔHbO_2_ [*F*(1) = 0.04, *p =* 0.840] and ΔHHb concentrations [*F*(1) = 0.6, *p =* 0.441], nor interactions between VMS and sessions [ΔtHb: *F*(2) = 0.5, *p =* 0.625; ΔHbO_2_: *F*(2) = 0.5, *p =* 0.583; ΔHHb: *F*(2) = 0.95, *p =* 0.394].

Concerning cognitive performance, no effect of VMS was observed [n‐back: *F*(1) = 0.9, *p =* 0.351; Stroop: *F*(1) = 0.5, *p =* 0.467; MEM‐III: *F*(1) = 0.9, *p =* 0.354], nor any interaction between VMS and sessions [n‐back: *F*(2) = 2.2, *p =* 0.126; Stroop: *F*(2) = 1.1, *p =* 0.344; MEM‐III: *F*(2) = 0.9, *p =* 0.424] or between VMS and time [n‐back: *F*(1) = 0.3, *p =* 0.628; Stroop: *F*(1) = 0.04, *p =* 0.840].

Likewise, VMS had no effect on prefrontal cortex oxygenation measures during the n‐back test [ΔtHb: *F*(1) = 1.3, *p =* 0.264; ΔHbO_2_: *F*(1) = 1.1, *p =* 0.313; ΔHHb: *F*(1) = 0.7, *p =* 0.416], Stroop task [ΔtHb: *F*(1) = 0.1, *p =* 0.738; ΔHbO_2_: *F*(1) = 0.06, *p =* 0.809; ΔHHb: *F*(1) = 0.1, *p =* 0.738] and MEM‐III [ΔtHb: *F*(1) = 0.3, *p =* 0.584; ΔHbO_2_: *F*(1) = 0.2, *p =* 0.628; ΔHHb: *F*(1) = 0.1, *p =* 0.734]. In the same way, no interaction effect was observed between time and VMS, nor between session and VMS for any of the parameters (ΔtHb, ΔHbO_2_ and ΔHHb) across cognitive tests.

#### 3.4.2 Effect of CRF

Regarding cerebrovascular responses to exercise, no effect of CRF was noted during the sessions [ΔtHb: *F*(1) = 0.07, *p =* 0.798; ΔHbO_2_: *F*(1) = 0.6, *p =* 0.447; ΔHHb: *F*(1) = 0.4, *p =* 0.540], nor any interaction between CRF and sessions [ΔtHb: *F*(2) = 0.7, *p =* 0.522; ΔHbO_2_: *F*(2) = 1.0, *p =* 0.365; ΔHHb: *F*(2) = 0.2, *p =* 0.800].

**FIGURE 5:**
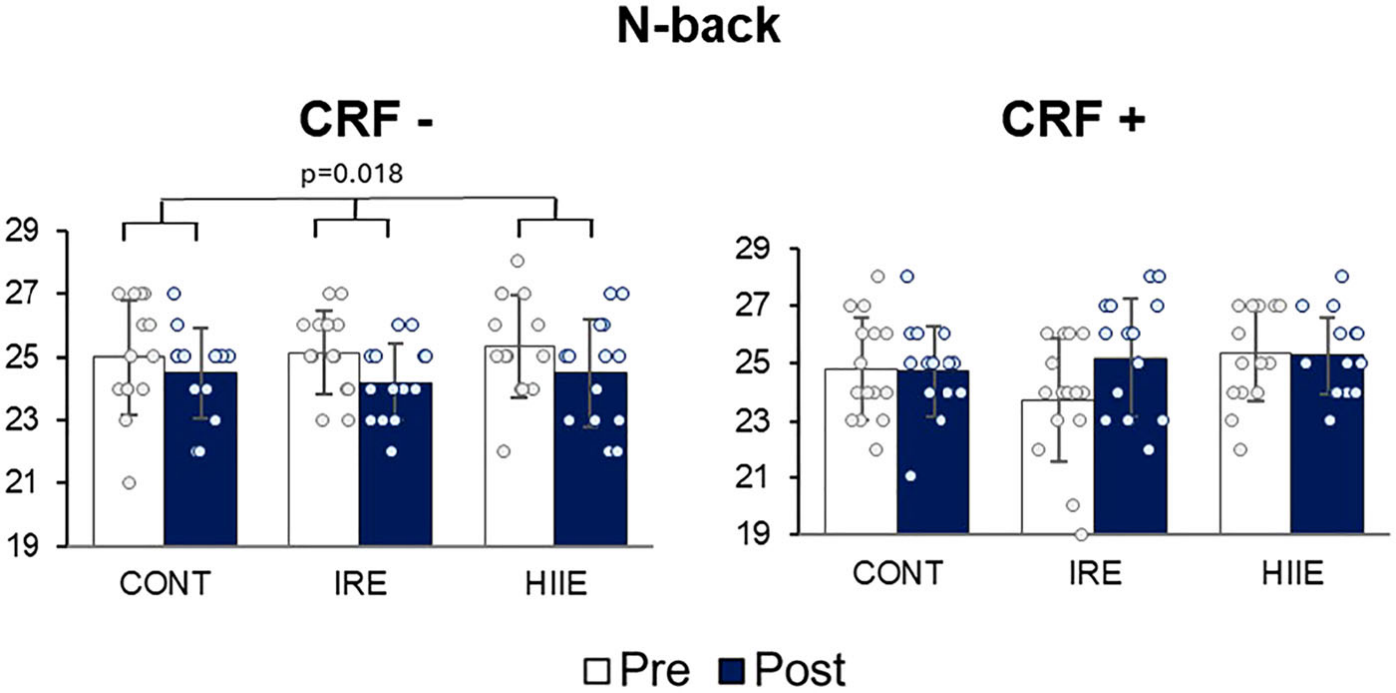
Influence of cardiorespiratory fitness on working memory response to sessions. ^*^ *p* < 0.05. Abbreviations: CONT, control session; CRF−, ${\dot{V}}_{O_{2}\max}$ ≤ 37.5 mL kg^−1^ min^−1^ (*n* = 14); CRF+, ${\dot{V}}_{O_{2}\max}$ > 37.5 mL kg^−1^ min^−1^ (*n* = 15); HIIE, high‐intensity aerobic intermittent exercise session; IRE, isometric resistance exercise session.

Concerning cognitive performance, no effect of CRF was observed [n‐back: *F*(1) = 0.01, *p =* 0.945; Stroop: *F*(1) = 0.5, *p =* 0.475; MEM‐III: *F*(1) = 0.9, *p =* 0.344], nor any interaction between CRF and sessions [n‐back: *F*(2) = 0.8, *p =* 0.459; Stroop: *F*(2) = 0.9, *p =* 0.392; MEM‐III: *F*(2) = 1.7, *p =* 0.201]. However, an interaction effect between time and CRF for the number of correct responses at the n‐back task was noted [*F*(1) = 14.4, *p <* 0.0001], but not for Stroop task [*F*(1) = 0.72, *p =* 0.403]. *Post hoc* analysis showed that cognitive performance decreased following the CONT, IRE and HIIE sessions in women with CRF− (*d* = 0.5, *p =* 0.018). In contrast, performance remained stable after these sessions in women with CRF+ (*d* = 0.3, *p =* 0.299). These results are shown in Figure 5.

Concerning cerebrovascular responses to exercise during cognitive tasks, no effect of CRF was observed for ΔtHb [n‐back: *F*(1) = 0.05, *p =* 0.835; Stroop: *F*(1) = 0.5, *p =* 0.476; MEM‐III: *F*(1) = 1.0, *p =* 0.322], ΔHbO_2_ [n‐back: *F*(1) = 0.4, *p =* 0.512; Stroop: *F*(1) = 0.9, *p =* 0.336; MEM‐III: *F*(1) = 2.2, *p =* 0.150] and ΔHHb concentrations [n‐back: *F*(1) = 0.2, *p =* 0.622; Stroop: *F*(1) = 0.03, *p =* 0.859; MEM‐III: *F*(1) = 0.01, *p =* 0.919], nor any interaction between session and CRF. However, a time × CRF interaction effect was observed on ΔHbO_2_ concentrations during the Stroop task [*F*(1) = 5.7, *p =* 0.025] and a trend for the n‐back test [*F*(1) = 3.97, *p =* 0.058]. For the Stroop task, women who were CRF+ exhibited a more pronounced cerebrovascular response to the sessions (pre vs. post: *d* = 1.13, *p <* 0.0001) in comparison to those who were CRF− (pre vs. post: *d* = 0.55, *p =* 0.027). A similar effect was observed for the n‐back test between CRF− (pre vs. post: *d* = 0.6, *p =* 0.022) and CRF+ (pre vs. post: *d* = 1.12, *p <* 0.0001) groups. In contrast, no interaction between time and CRF for ΔHHb and ΔtHb was noted.

## 4 DISCUSSION

The primary aim of this randomized controlled cross‐over study was to investigate the impact of two distinct exercise modalities (IRE and HIIE) on different high‐level cognitive functions (episodic memory, inhibitory control and working memory) and prefrontal cortex oxygenation in postmenopausal women. Additionally, the study sought to explore whether these responses were influenced by the severity of VMS or the CRF of participants. The findings revealed no significant effect of acute exercise on cognitive performance. However, increases in prefrontal cortex oxygenation were observed during both the IRE and HIIE sessions and after the HIIE session across all cognitive tasks. Notably, CRF emerged as a significant factor modulating cognitive and cerebrovascular responses, whereas no influence of VMS was observed.

### 4.1 Acute effect of physical exercise on neurocognitive responses

The lack of improvement in executive functions following the exercise sessions contrasts with the findings of the meta‐analysis by Moreau and Chou (2019), which showed that a session of HIIE has a positive effect on inhibitory control [*d* = 0.27 (95% confidence interval 0.08; 0.46)] and working memory updating [*d* = 0.32 (95% confidence interval 0.08; 0.55)] (Moreau & Chou, 2019). However, this meta‐analysis indicates that the positive effects of HIIE are observed primarily in studies involving participants aged 19–30 years. This finding is supported further by the systematic review by Hsieh et al. (2021), which examined the impact of HIIE on executive functions across different age groups (Hsieh et al., 2021). Hsieh et al. (2021) concluded that the acute benefits of HIIE are seen primarily in children, adolescents and young adults, with the effects on middle‐aged and older adults requiring further investigation. Likewise, the observed benefits of IRE on executive functions in the literature are largely based on studies involving young adult populations (Zhu et al., 2022).

In our study, we did not find a positive effect of either HIIE or IRE on episodic memory function in postmenopausal women. The meta‐analysis by Loprinzi et al. (2019) demonstrated that the acute benefit of physical exercise on episodic memory varies depending on the timing of the exercise relative to the retention task. They assessed the effectiveness of exercise based on whether it was performed before encoding (e.g. before the two story presentations of the MEM‐III), during encoding (between the two readings), during the early consolidation phase (after the presentations but before the delayed recall, as in our case) or during the late consolidation phase (delayed recall several hours after exercise). Their results indicated that the benefits are enhanced when exercise is performed during the early and late consolidation phases. However, the positive effect observed during the early phase is limited to young adults aged 18–24 years, which aligns with the results of our study (Loprinzi et al., 2019).

Although we did not observe an effect of exercise sessions on cognitive responses, a positive effect on cerebral oxygenation was noted. In fact, our study showed a significant increase in oxygen delivery to the prefrontal cortex (ΔHbO_2_) during both exercise sessions compared with the CONT session (IRE: *d* = 0.89; HIIE: *d* = 0.99) and a significant increase in ΔHHb (*d* = 0.68) and ΔtHb (*d* = 0.96) during the HIIE session. Comparable results were reported by Monroe et al. (2016), who observed an increase in oxyhaemoglobin and deoxyhaemoglobin concentrations during a HIIE session compared with continuous exercise (Monroe et al., 2016). Likewise, Keller et al. (2022) found an increase in prefrontal cortex oxygenation during IRE exercise at 25% of MVF in young women (Keller et al., 2022). After the HIIE session, concentrations of ΔHbO_2_ and ΔtHb increased significantly (*d* > 1) in comparison to the measures before the session and after the IRE and CONT sessions. These data suggest that blood flow remains elevated after the HIIE session, potentially promoting cerebrovascular adaptations that could be reinforced by repeated exposure to high‐intensity interval training (Calverley et al., 2020).

### 4.2 Influence of VMS on neurocognitive responses to physical exercise

Our initial hypothesis was that the severity of VMS might negatively influence neurovascular responses to exercise. This assumption was based on literature suggesting that women with VMS experience impaired hippocampal function and dysfunction in several regions of the prefrontal cortex (Maki et al., 2020). Moreover, VMS have been linked to poorer cardiovascular health, including compromised vascular function (Bechlioulis et al., 2010), which might impair cerebrovascular responses, such as cerebral perfusion (Barnes & Corkery, 2018). Given these vascular changes, we expected that women with moderate to severe VMS would exhibit poorer cognitive performance, particularly in tasks involving prefrontal cortex function. However, the present study did not reveal any effect of VMS on cognitive performance (at rest or after exercise) or on prefrontal cortex oxygenation. Our results contrast with studies showing a negative impact of VMS on episodic memory (Maki et al., 2008, 2020). However, these studies measured VMS objectively using skin conductance, whereas our study relied on subjective self‐reports via questionnaires, which could account for the discrepancy. Indeed, the validity of self‐reported VMS has been questioned in previous studies, primarily because the presence of other menopausal symptoms (such as sleep disturbances and mood disorders) might impact the perception of VMS, and secondly, owing to recall bias, particularly with nocturnal VMS (Maki & Thurston, 2020). Concerning executive functions, including working memory and inhibitory control, our findings align with previous research showing no significant association between subjective VMS and working memory performance (Debray et al., 2022; Weber et al., 2013).

### 4.3 Influence of CRF on neurocognitive responses to physical exercise

Previous studies have reported positive effects of CRF on cognitive and cerebrovascular responses, both at rest and during exercise (Rigdon & Loprinzi, 2019; Salzman et al., 2022), including in postmenopausal women (Peiffer et al., 2015). In our study, we did not find an increase in cognitive performance after exercise sessions. However, our study highlights a beneficial role of CRF in maintaining working memory updating performance. Specifically, women with lower fitness levels (${\dot{V}}_{O_{2}\max}$ ≤ 37.5 mL kg^−1^ min^−1^) experienced a moderate decline in cognitive performance between the beginning and end of sessions, probably owing to reduced attentional resources over time. In contrast, women with higher fitness levels (${\dot{V}}_{O_{2}\max}$ > 37.5 mL kg^−1^ min^−1^) maintained stable performance throughout, suggesting that greater CRF helps to sustain cognitive performance during extended tasks. This is consistent with prior research indicating that reduced CRF can impair attentional capacity during demanding tasks (Luque‐Casado et al., 2016).

Additionally, a significant difference in prefrontal cortex oxygenation was observed during cognitive tests following the sessions. Highly fit women (${\dot{V}}_{O_{2}\max}$ > 37.5 mL kg^−1^ min^−1^) exhibited a larger cerebrovascular response (large effect size) in comparison to those with lower CRF (moderate effect size). This finding points to enhanced cerebral perfusion in women with greater CRF, supporting evidence from Salzman et al. (2022) that links higher fitness levels to improved cerebrovascular function (Salzman et al., 2022). Together, these results reinforce the notion that CRF plays a crucial role in sustaining cognitive and cerebrovascular health, particularly during and after physical activity.

### 4.4 Strengths and limitations

Our study had several notable methodological strengths. The randomized controlled crossover design minimized the influence of confounding factors, because each participant served as her own control. Furthermore, sessions were conducted at the same time of day on similar days to ensure comparable attention levels within participants, which helped to minimize the impact of circadian variation on cognitive function, as supported by previous research (Valdez, 2019). Additionally, to ensure consistency in exercise intensity, sessions were personalized based on the fitness level of each participant. Maximal exercise tests (${\dot{V}}_{O_{2}\max}$ and MVF) were performed before the exercise sessions to access individual capacity and adjust exercise intensity, thereby ensuring similar relative intensity across participants.

Moreover, we chose to calculate the CRF based on lean mass rather than total mass, to exclude fat mass from the calculation and to optimize the accuracy of the physical fitness classification (CRF−/CRF+). Nevertheless, when expressed per total body mass, the mean CRF of our population was 25.8 ± 6.3 mL kg^−1^ min^−1^, which aligns with the reported mean of 27.6 ± 9.1 mL kg^−1^ min^−1^ for middle‐aged women (Myers et al., 2017). In addition, the mean CRF per total body mass was 21.3 ± 2.7 mL kg^−1^ min^−1^ in the CRF− group and 30.7 ± 5.3 mL kg^−1^ min^−1^ in the CRF+ group. Therefore, our median split effectively distinguishes two groups with different CRF. Furthermore, the different groups (VMS−/VMS+ and CRF−/CRF+) were homogeneous in their characteristics, except for the main discriminant variable (CRF or VMS), ensuring that any differences observed could be attributed to the specific factors under investigation. Finally, none of the women in this study was on menopausal hormone therapy, which ensured a consistent hormonal status among the participants, which might otherwise have influenced cognitive and cerebrovascular responses to exercise.

However, our study has several limitations. One of these is the lack of activity homogenization during the control sessions. Although participants were instructed to choose activities with minimal cognitive load, variability in choices might have introduced inconsistencies. Additionally, the two exercise modalities chosen (HIIE and IRE) differed in Metabolic Equivalent of Task and duration. They were selected for their contrasting modes of contraction, ease of implementation, and time efficiency, because time constraints are a common barrier to exercise in postmenopausal women (Wasley & Gailey, 2024).

Secondly, for the VMS classification, the use of the MRS questionnaire (subjective assessment) to differentiate between women with and without moderate to severe VMS might have influenced the results of our study regarding the effect of VMS on cognitive response to exercise. As mentioned above, several studies have found different effects of VMS on cognitive performance depending on the method of symptom assessment (objective vs. subjective) (Maki et al., 2008, 2020), because subjective measures might be susceptible to recall bias and influenced by other symptoms on VMS perception (Maki & Thurston, 2020).

Additionally, although inter‐individual covariates (e.g. age, body mass index, MoCA score, education level) were not included as covariates in our statistical analyses, which represents a limitation, the groups did not differ on these variables, and their minimal variability limits their impact on neurocognitive outcomes. Therefore, the homogeneity of our population is also a limitation, because our results might not be generalizable to all postmenopausal women, particularly those with high body mass index or poorer cognitive health.

Concerning cognitive tests, a potential limitation is the practice effect, because the repeated‐measures design and interval between sessions (10 ± 6 days) might have improved performance independently of the interventions. However, our crossover design minimizes this effect, with equivalent pre‐test scores across sessions (Table 2). In addition, the use of paper and pencil cognitive tests rather than a computerized version restricted our ability to measure additional cognitive response indicators, such as reaction time or accuracy percentage on the Stroop task. Finally, our participants had an average MoCA score of 27.7 ± 1.2 (range 26–30), reflecting a good overall cognitive level. This high initial performance might have limited the observed effects, because a higher proportion of participants with mild cognitive impairment (scores between 19 and 26) might have led to more pronounced results (Nasreddine et al., 2005). Likewise, high baseline cognitive performance was observed both before the sessions and during the control sessions (Table 2), which might have limited further the possibility of improving performance through physical exercise owing to a ceiling effect (Zorowitz & Niv, 2023).

## 5 CONCLUSION

This study shows that an IRE or HIIE session does not directly improve cognitive performance in postmenopausal women, although it does increase prefrontal cortex oxygenation, particularly after HIIE. CRF appears to play a role in postexercise cognitive and cerebrovascular responses, in contrast to vasomotor symptoms. These results highlight that VMS severity, although challenging, does not determine cognitive responses to exercise, emphasizing the importance of maintaining a high CRF to promote brain health in this population.

## AUTHOR CONTRIBUTIONS

Morgane Le Bourvellec, Nathalie Delpech, Laurent Bosquet, Geoffroy Boucard and Carina Enea conceived and designed the research. Morgane Le Bourvellec collected and analysed the data. Morgane Le Bourvellec, Nathalie Delpech, Geoffroy Boucard and Carina Enea interpreted the experimental results. Morgane Le Bourvellec prepared the figures; Morgane Le Bourvellec, Nathalie Delpech and Carina Enea drafted the manuscript Morgane Le Bourvellec, Nathalie Delpech, Laurent Bosquet, Geoffroy Boucard and Carina Enea approved the final version of the manuscript and agree to be accountable for all aspects of the work in ensuring that questions related to the accuracy or integrity of any part of the work are appropriately investigated and resolved. All persons designated as authors qualify for authorship, and all those who qualify for authorship are listed.

## AKNOWLEDGEMENTS

The authors would like to thank all the study participants, in addition to the study contributors: Dr Jaqueline Le Hennaff for supervising the maximal effort tests, and Melissa Demion for assisting with the sessions. The authors would also like to thank the BIO 86 biochemistry laboratory for conducting the blood sample analyses.

## CONFLICT OF INTEREST

None declared.
